# Bilateral, Misalignment-Compensating, Full-DOF Hip Exoskeleton: Design and Kinematic Validation

**DOI:** 10.1155/2017/5813154

**Published:** 2017-07-16

**Authors:** Karen Junius, Marc Degelaen, Nina Lefeber, Eva Swinnen, Bram Vanderborght, Dirk Lefeber

**Affiliations:** ^1^Department of Mechanical Engineering and Flanders Make, Vrije Universiteit Brussel (VUB), Pleinlaan 2, 1050 Brussels, Belgium; ^2^Department of Physical Education and Physiotherapy Rehabilitation Research, Vrije Universiteit Brussel (VUB), Laarbeeklaan 103, 1090 Brussels, Belgium; ^3^Rehabilitation Hospital Inkendaal, Inkendaalstraat 1, Vlezenbeek, 1602 Sint-Pieters-Leeuw, Belgium

## Abstract

A shared design goal for most robotic lower limb exoskeletons is to reduce the metabolic cost of locomotion for the user. Despite this, only a limited amount of devices was able to actually reduce user metabolic consumption. Preservation of the natural motion kinematics was defined as an important requirement for a device to be metabolically beneficial. This requires the inclusion of all human degrees of freedom (DOF) in a design, as well as perfect alignment of the rotation axes. As perfect alignment is impossible, compensation for misalignment effects should be provided. A misalignment compensation mechanism for a 3-DOF system is presented in this paper. It is validated by the implementation in a bilateral hip exoskeleton, resulting in a compact and lightweight device that can be donned fast and autonomously, with a minimum of required adaptations. Extensive testing of the prototype has shown that hip range of motion of the user is maintained while wearing the device and this for all three hip DOFs. This allowed the users to maintain their natural motion patterns when they are walking with the novel hip exoskeleton.

## 1. Introduction

Exoskeletons are usually divided in three classes depending on their aimed use: performance augmentation, rehabilitation, and assistance [1, 2]. A shared design goal for most robotic lower limb exoskeletons is to reduce the metabolic cost of locomotion for the user [3, 4]. Despite this common goal, only a limited amount of devices was able to reduce the metabolic consumption of the user during powered walking [5]. Most of these are tethered and because the weight of the device has a significant impact on the energy consumption of its wearer [6–8], they usually do not span the entire lower limbs [9–11]. When exoskeletons are meant for assistance of activities of daily life, for example, assisting the elderly or allowing paraplegics to walk, being tethered is unacceptable, while the reduction of user energy consumption remains vital [12]. Only recently were researchers capable of reducing user effort with autonomous, untethered exoskeletons [13, 14]. The devices used in these two studies are meant to assist the ankle joint of the wearer during the push-off phase in walking.

Asbeck et al. [12] stated that for an assistive device to be metabolically beneficial, it should apply the right amount of assistance at the right time to the body, as well as maintain the normal biomechanics of motion and minimize additional mass carried by the wearer, particularly on the distal portion of the leg. Taking these three requirements into account, the use of a full-DOF (degree of freedom), misalignment-compensating hip exoskeleton appears to be a suitable tool to achieve this. Although actuating the ankle joint is a reasonable strategy, the hip joint is also seen as a significant contributor of positive work [9, 15] and it is less dependent on passive mechanisms, thus powering it may be metabolically more efficient [3, 16]. Additionally, it is less susceptible to the addition of mass than the ankle [7, 17, 18] and maintaining unrestricted hip motion is considered important for the energy efficiency of gait [19, 20]. Because the “right” assistance is highly dependent on the physical build of the user and his/her affliction, this paper will focus on the preservation of natural biomechanics by not restricting motion of the hips.

Despite the argumentation above, most state-of-the-art exoskeletons still incorporate single DOF hip joints that need to be manually aligned with the human joint [21–23]. Alignment of exoskeleton and biological axes of rotation is vital for a correct transmission of torque from actuator to human [24]. If not perfectly aligned, significant disturbance forces on the wearer can be created, causing discomfort, pain, and even injury [25]. Ironically, perfect alignment is an unattainable state. This is due to a combination of joint coverage, large inter- and intrasubject variability, modeling approximations, and slippage of the exoskeleton during use [24, 26, 27]. Only a limited amount of research groups has already invested resources into research in multi-DOF hip joints with some sort of misalignment compensation mechanism. Bartenbach et al. [28] created a bilateral hip and knee exoskeleton with sliding brace structures to allow the relative movement between exoskeleton and human caused by misalignment. However, these structures are only qualified to alleviate misalignment complications of the 1-DOF knee joint. In order to maximize kinematic compatibility, a 3-DOF hip with intersecting axes was developed, thus making it possible to reduce but not eliminate misalignment of the hip DOFs. Beil and Asfour [29] did aim to eliminate misalignment issues in the hip joint, yet only for the rotational DOF. The prototype consists of a back connection with sequentially an abduction axis, a rotation mechanism, and a flexion axis that is connected to the thigh. The abduction and flexion axes each consist of a single hinge and need to be manually aligned with the human axes, while the rotational mechanism consists of three hinges and two sliders and is self-aligning. In [29], the range of motion (ROM) of the prototype is considered to be sufficient to allow its wearer to walk unhindered, yet it is also remarked that due to flexible deformation of the “rigid” structures, the results might be influenced. Note that an expansion of this principle to a 3-DOF system would result in a complex device, requiring nine hinges and six sliders per hip. In [30], an exoskeleton with DOF hips is presented, where misalignment of the flexion axes is compensated. Although the abduction axes are not aligned, nor is misalignment compensated, Giovacchini et al. indicate that this is not seen as a hindrance by the user. A last device was developed as a part of the Robo-Mate project and incorporates a pelvis module with 2-DOF hip joints [31]. Each side of the module consists of an active flexion/extension joint, two passive abduction axes and a passive ball joint that connects the thigh brace to the frame. The second abduction joint in combination with the ball joint connection to the brace is said to cope with misalignment effects, yet this is hard to evaluate due to the limited ROM of the device.

The novelty of the contributions in this paper is the design of a lightweight and compact misalignment compensation mechanism for a 3-DOF system, which is validated by a hip exoskeleton prototype. As such, this allows for the wearer of the exoskeleton to move around unhindered by the device and thus maintain his natural gait kinematics and full biological hip ROM. Chapter II describes the requirements of the hip exoskeleton resulting from the anatomy of the human hip joint. In chapter III, the design choices are explained. Chapter IV gives an overview of the equipment, experimental protocol, and data analysis techniques that were used to validate the novel prototype. A maximum ROM study is performed and used as a guideline to make conclusions that are valid for activities of daily life. The validity of this extrapolation is verified by analysing the gait cycle during level overground walking. The results of this validation process are presented in chapter V and discussed in chapter VI. Conclusions and future work points are formulated in chapters VII and VIII.

## 2. Design Requirements

### 2.1. Criteria of User Acceptance

The final aspiration of all exoskeleton research is the design of a device or a mechanism that is useful in its aimed application. In the pursuit of solving complex technological difficulties, user acceptance is an important criterion that is often forgotten in the design process. This is unfortunate because although assistive devices can have a profound effect on a person's abilities, such devices generally have a high abandonment rate [32]. Given the influence that user opinion has on the adoption of an assistive device, the authors felt it was important to include user acceptance criteria in the evaluation of a preliminary design. Important influences on nonuse of assistive devices are related to their appearance and ease of use [33–35]. Translating this to the design requirements calls for a device that is lightweight, close to the body, requires short donning time, and is suited for autonomous donning. Another significant factor associated with continuance or discontinuance of technology is the relative advantage of the device [36]. Important criteria that users utilize to assess the relative advantage are comfort, safety, effectiveness, and durability [37, 38].

### 2.2. Hip Joint Anatomy

**Figure 1:**
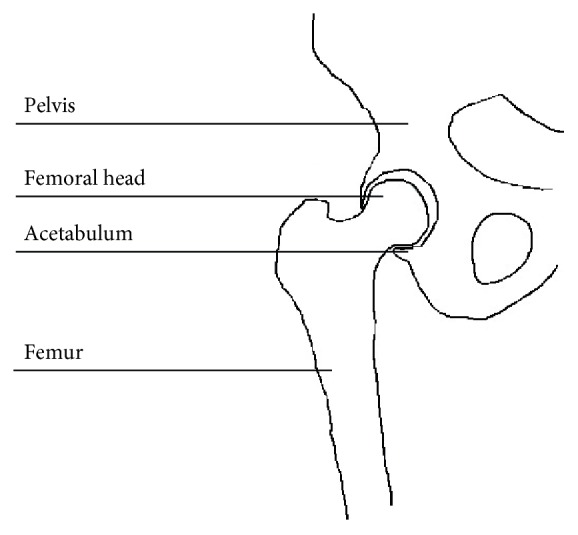
Human hip joint anatomy.

In order to allow unhindered motion, the exoskeleton needs to be kinematically compatible with the human hip joint [39]. The hip represents the junction between the pelvis and the femur and is shown in Figure 1. The actual connection is achieved between the top of the femur, that is, the femoral head, and a cup-shaped zone on the pelvis, that is, the acetabulum, by an extensive set of connective tissues and muscles. It shows close resemblance to a ball and socket joint, where the femoral head represents the ball and the acetabulum represents the socket. Ideally, the exoskeleton hip joint should thus be three dimensional and its center of rotation should coincide with the hip joint center. However, the location of the hip joint center is not easily determined due to joint coverage [40]. Several methods are available to determine the location of the hip joint center, yet predictive methods using the location of certain bony landmarks remain the only clinically feasible method [41]. An added difficulty lies in the joint's protection mechanism from damage due to dangerously high contact forces: when the hip is loaded, for example, during single-leg stance, the acetabulum deforms, increasing the contact area with the femoral head [42, 43]. This causes a small movement of the femoral head with respect to the pelvic landmarks that are used to determine the location of the hip joint center. Due to the variability of biomechanical parameters between subjects, for example, shape and density of the bones, and the variability of some parameters within individual subjects during movement, for example, load conditions, it is impossible to correctly predict the motion of the hip joint center of an individual [44, 45]. Thus, compatible kinematics, leading to a high-quality interaction, are best approached by introducing redundant joints into the exoskeleton design, rather than trying to exactly match human joints [45].

### 2.3. Compensation of Misalignment

As it is impossible to exactly match the kinematics of human joints, for reasons described in Section 2.2, often researchers fall back to simplified models [46]. In locomotion analysis, the human joints are routinely modeled as a set of hinges oriented along the intersections of the sagittal, frontal, and transverse plane [47, 48]. As described earlier, the hip joint is three dimensional, thus requiring all three. Correct alignment of exoskeleton hinges with biological axes of rotation is crucial to ensure the correct transfer of torque from exoskeleton to wearer [24, 40]. For the hip, this means the intersection of the three axes at the hip joint center, which is shown to be nearly impossible in the previous section. Errors of 8–13 mm between the estimated and actual hip joint center are considered a good approximation [49, 50]. Even in the unlikely case that close alignment is achieved, the links between the user and the device are likely to slip during operation [26, 51–53], due to soft tissue deformations [44]. This is particularly relevant for the hip joint which relies, due to its anatomy, on a multitude of tissue and muscle structures to guarantee joint stability [43]. It is thus safe to conclude that perfect alignment of all three hinge joints with the biological axes of rotation is unattainable.

Consequences of misalignment are severe: parasitic torques of up to 1.5 Nm have been documented due to misalignment and this in absence of any actuation [54]. This can cause discomfort or pain and may potentially even lead to long term injury or dislocation of the joint [45, 52, 55–57]. It is thus imperative that misalignment effects are compensated for, and this for all three DOFs.

In most existing exoskeletons, where manual alignment of the joint axes is still executed, the assumption is made that any change in angle of the exoskeleton joint corresponds to an identical change in angle of the human one. This simplifies the design of the actuation system a grave deal, as the state of the human can at any time be determined by measurement of the exoskeleton variables; for example, angles and speeds. Therefore, there is no need for the placement of sensors on the human limbs, reducing the mass and the complexity of the system. The same should be strived for in the misalignment compensation mechanism.

### 2.4. Requirements

As summarized, the previous subsections lead to the following set of design requirements:

- 3 perpendicular rotation axes
- Misalignment compensation mechanism for all axes
- Lightweight
- Well fixated, yet comfortable interface
- Use of robust elements with low maintenance rate
- Low donning time
- Autonomous donning possible
- Compact that is not protruding
- Equal angle changes.

## 3. Design of the Hip Exoskeleton

### 3.1. Principle

**Figure 2:**
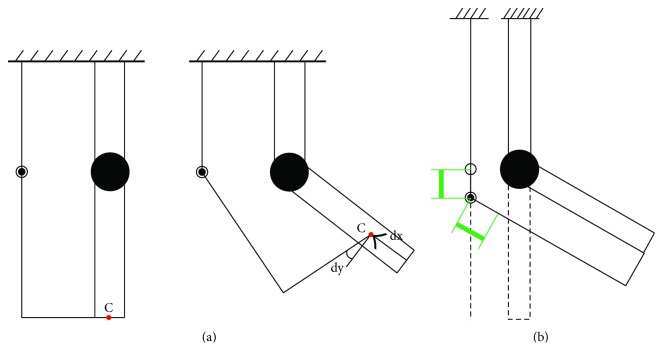
Human-robot interaction in the case of a single DOF human and exoskeleton joint.

The design of the novel hip exoskeleton was based on a basic principle of mechanics: the parallel axis theorem. This states that the displacement of a rigid body due to a pure rotation about any line is equivalent to a displacement due to an equal rotation about a parallel line together with a translation perpendicular to that line [58]. Applying this in exoskeleton design would mean that any rotation around a biological axis is identical to a rotation around the corresponding exoskeleton axis, if a translation of the exoskeleton axis in the plane perpendicular to it is allowed. Using a 1-DOF joint as an illustrative tool, this could be achieved by allowing a change in length of the two joint links. In Figure 2(a), a single joint exoskeleton limb and a single joint human limb are shown. The upper links of both limbs are grounded. The lower link of the exoskeleton is connected to the human limb by means of a cuff, represented by the connection point C. When the human limb rotates, the exoskeleton follows. Yet, misalignment of the joints results in a movement, dx, of the connection point along the human arm. Additionally, the exoskeleton rotates with respect to the human limb over an angle dj. When the exoskeleton links are allowed to change length, as shown in green in Figure 2(b), the hinge translates in the plane perpendicular to the rotation axis. The connection point C is not influenced during a rotation of the human arm. It is also important to notice that the exoskeleton hinge exhibits the same angle change as the human joint.

### 3.2. Implementation

**Figure 3:**
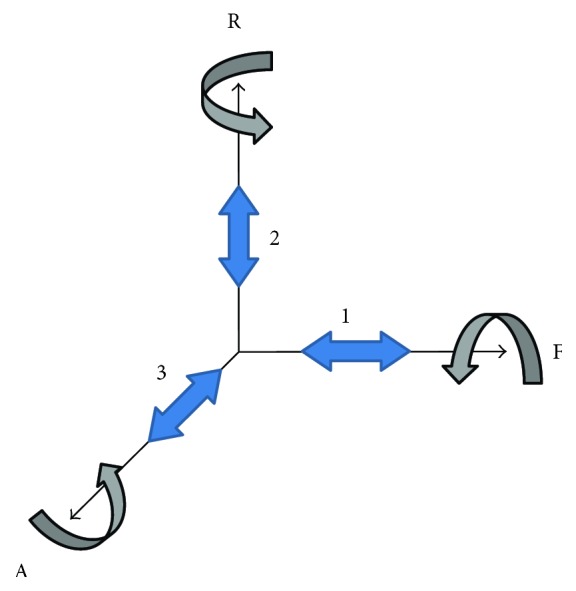
Required elements for misalignment compensation in three dimensions. The misalignment compensation mechanism of any of the rotational DOFs consists of the sliders perpendicular to the rotation axis. For flexion/extension (F), movement of sliders 2 and 3 is required; for internal/external rotation (R), sliders 1 and 3; and for abduction/adduction (A), sliders 1 and 2.

Incorporating a misalignment compensation mechanism for each of the axes of the 3-DOF hip joint would, by extrapolation, require three hinges and six linear sliders. However, by utilizing three perpendicular sliders, each one can act as a functional part of two misalignment compensation mechanisms. In Figure 3, the three required rotational hinges are shown. One allows flexion/extension, another abduction/adduction, and the last internal/external rotation and are denoted by, respectively, F, A, and R. The three perpendicular sliders are each positioned along one of the hinges, that is, at location 1, 2, and 3. Misalignment compensation, for each of the rotational DOFs, is then provided by movement of the sliders perpendicular to the rotational axis. This way, slider 1 aids in the compensation of R misalignment as well as A misalignment. Analogously, slider 2 aids in F and A misalignment compensation and slider 3 in R and F misalignment compensation.

Following the reasoning above, for one full-DOF, misalignment-compensated hip joint, six elements are required: three rotational hinges and three linear sliders. The exoskeleton is meant to operate in parallel with the human hip joint, so it is connected to the user at the torso and at the thigh. All six joint elements need to be fitted in between these connections. Note that the sequence of sliders and hinges is of no importance for the functionality of the 3-DOF joint. This implies that all of the 720 (=6!) different sequences are a viable option from a mechanical point of view. The suitability of each of these thus depends on criteria that are characteristic for the application. By demanding that elements are positioned along the human in such a way to limit protrusion but not cause collision, that none of the elements are unnecessarily positioned on the thigh, and that the effect of gravity on the abduction hinge is taken into account, the number of possible sequences is reduced to six. At this point, a comparison strategy was followed, scoring each of the remaining possibilities on compactness, arm swing obstruction, weight location, and required slider motion. A value between 1 and 5 was attributed to each of the remaining sequences, indicating their performance in each of the abovementioned qualities. A score of 1 represents that the sequence is not compact at all, no arm swing is possible at all, weight is all on the thigh, and slider motion is attaining a maximum value. Analogously, a score of 5 represents the other end of the spectrum, where each of the qualities is as ideally as expected. The sequence with the highest sum of scores was used as a template for the design of the actual prototype.

**Figure 4:**
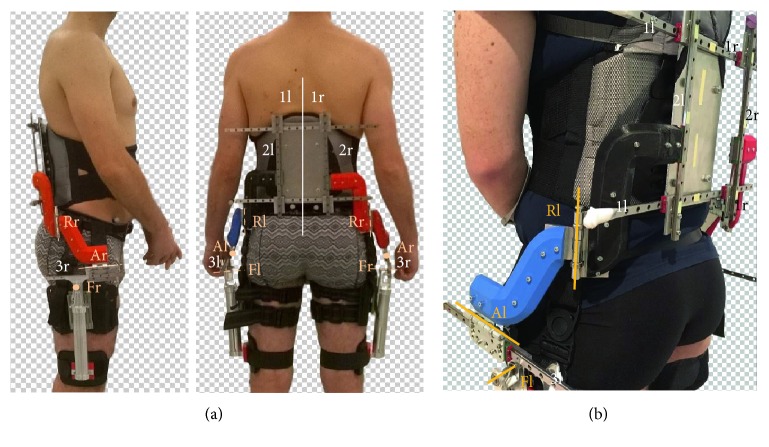
Bilateral, full-DOF, misalignment-compensating hip exoskeleton. Linear sliders are numbered in white, and rotational joints are marked and named in orange. The added suffix l or r indicates the use of each element by the left of right hip. (a) Overall side and back view. (b) Close-up of the left side of the prototype.

In Figure 4, the final prototype is shown. The overall back and side views are given in Figure 4(a), and a close-up of the left side is provided in Figure 4(b). The sliders and rotational joints are numbered and named to highlight the sequence that was selected in the procedure discussed above. For clarity, the same letter and number code was used as described in Figure 3. The sequence of elements starts with the horizontal 1-sliders that are mounted onto the rigid metal plate and the vertical 2-sliders that can slide on the former. Both are responsible for motion in a plane parallel to the back and can thus be mounted in close proximity to the user. This not only favors compactness of the design but also ensures a mass distribution close to the human's center of mass and is thus metabolically favorable. Note that the 1l- and 1r-sliders are in fact not two separate sliders, but the left and right side of the two parallel 1-sliders. This because it decreases the load on the individual sliders and ensures better torque transmission onto the user. Next in the sequence is the R-axis, which is situated in between the user's back and side. This location was selected because it limits the offset between the exoskeleton and human rotation axes and thus limits slider motion for misalignment compensation. On the side of the user, more or less at the height of the biological hip joint, the A-axis, 3-slider, and F-axis are positioned in the given order. The side location was deemed most appropriate to ensure device compactness, reduce the influence of gravity on the A-axis, and limit the offset from the biological axes and thus slider travel. Localization around the biological joint level was considered a good trade-off between loading of the thigh and obstruction of arm swing. The sliders and axes are connected to each other by rigid metal parts and 3D-printed components that are shaped in such a way as to provide efficient torque transfer while maintaining compactness. The connections from the 2-slider to the R-axis (black for the left side and red for the right side) and from the R-axis to the A-axis (blue for left and red for right side) curve close around the human body, leaving a small gap that is sufficiently large to prevent collision of the exoskeleton with the human.

The exoskeleton axles are realised with ball-bearing hinges from Breuer & Schmitz that do not require greasing and are not sensitive to dirt. The same applies for the sliders, which are from the Hiwin MGN series and are able to withstand the assistive torques that the prototype will be providing in a next phase of the project. The connection to the user was done with commercially available systems. The connection around the torso is a Dainese WAVE kidney belt, commonly used as protection in motorcycle sports. It has a reinforced back structure that allows for a rigid connection to the exoskeleton and is easily fitted onto a wide range of subjects due to the Velcro strap. Tight fitting of the strap prevents the belt from sliding down as it rests in the smallest zone of the users' waist. The connection to the thigh is realised by two separate straps: one just below the groin and the other above the knee joint. Both are repositionable on the thigh to increase user comfort. The bottom one is a conventional Velcro pull-eye strap, with a width of 5 cm. A rectangular, flexible pad was added to the side of the thigh to increase the contact zone in between the exoskeleton and user and decrease the risk of chafing and so on. The top connection is derived from a thigh holster, which is secured around the thigh as well as around a hip belt, to prevent sliding down of the holster along the leg. This is particularly important for this prototype as the presence of the vertical 2-sliders allows for such a motion to happen. All elements included the device that weighs approximately 4.8 kg, of which 1 kg is mounted at the side of the user, on each of the thighs. A reduction of 200 g of the total mass is possible by decreasing slider length. Length of the sliders was overestimated in order to prevent ROM limitation due to insufficient linear travel range.

### 3.3. Operation of the Design

**Figure 5:**
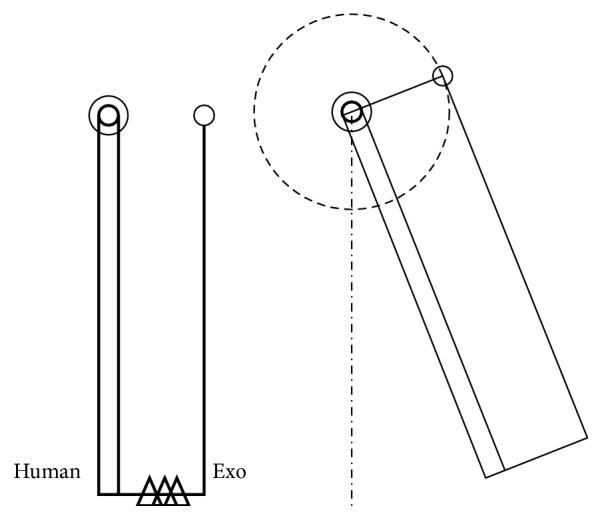
Schematic representation of human-exoskeleton interaction during abduction. Considering that the human hip joint is the driving force of the motion, abduction of the hip causes a rotation of both the thigh and the exoskeleton around the human abduction axis.

In order to clearly show the operating principle of the design, a schematic representation of abduction is given in Figure 5. In this figure, the two concentric circles represent the hip joint projected onto the frontal plane. Attached to it is the thigh, represented by the rectangle. On the right side of the hip and thigh are the corresponding exoskeleton joint and limb. The exoskeleton limb is connected to the thigh through a strap at the bottom, represented by the crossed line. In reality, the human is driving the motion and the passive exoskeleton follows along with that motion. Therefore, in this example, the human hip center is considered fixed. Assuming that the connection between the human and the exoskeleton is rigid, abduction of the human hip results in an identical rotation of the human thigh, as well as the exoskeleton, around the biological abduction axis. During this motion, the exoskeleton joint follows a circular path around the human joint axis. Allowing this motion maintains the distance and orientation between the thigh and exoskeleton limb, thus eliminating misalignment effects.

**Figure 6:**
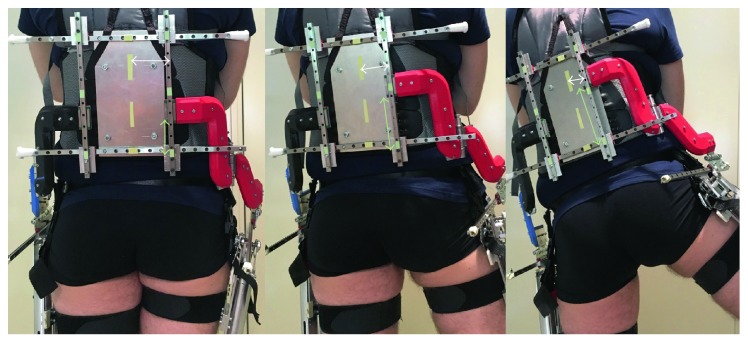
Abduction of the right leg, while wearing the novel prototype. As soon as the leg swings outwards, the vertical slider is pushed upward, as seen by the increasing green arrow. Additionally, the horizontal slider starts to move as well: it is pushed towards its center, visualised by the decreasing white arrow.

In Figure 6, the real life scenario is shown. In the photo series, one of the subjects is abducting the right hip. As the leg is moving outward, the vertical slider is moving upward. This is visualised in the figure by the increasing distance between the bottom 1-slider and the red connection piece, marked in green. Simultaneously, the horizontal slider is slowly moving towards its center. The distance between the slider position and the center is marked in white. Note that these movements of the sliders, that is, up and in, correspond to the circular path that was shown in the schematic representation.

## 4. Method

In this section, the method, to validate the novel hip exoskeleton, is discussed. A maximum ROM determination was performed first. These measurements can be used to form a first conclusion on the executability of other activities of daily life while wearing the prototype, considerably reducing required testing times. The validity of using maximum ROM data to form these conclusions is tested by evaluating overground gait kinematics. Tests are performed three times: unequipped (UE), wearing the novel prototype (PT), and wearing a 1-DOF hip exoskeleton (1D) that needs to be manually aligned (representing the current state of the art).

### 4.1. Subjects

Since the experiment serves as a mere proof of concept rather than a clinical trial, three test subjects were deemed sufficient. All subjects were young and healthy adults that volunteered for the study. Neither of them were, at the moment of the test, being treated for any injury or affliction that influences ROM of the hip or interferes with gait. Data for each of the approved subjects is shown in Table 1. Subjects were selected based on height (>1 m70) and waist-to-hip ratio (0.8 < WHR < 1), to facilitate the test procedure. Subjects with a lower WHR are curvier, thus require different connection pieces in between the functional components to keep the prototype in close proximity of the body. Analogously, smaller subjects need shorter connection pieces because distance between the waist and the hip joint is smaller.

Instead of continuously changing connection pieces based on body shape, the selection of subjects based on the existing prototype was preferred. All subjects have provided a written consent to their participation to this study.

### 4.2. Equipment States

During the experiment, three different equipment states were investigated, that is, wearing the prototype (PT), unequipped (UE), and wearing a 1-DOF exoskeleton (1D). The new prototype was extensively discussed in Section 3.2 and is shown in Figure 4. For the 1-DOF exoskeleton, a hip module was used that was previously developed as a part of the MIRAD project [59]. The module consists of a rigid structure that is adaptable for hip width of the wearer, which is connected to a torso brace for connection to the user. A flexion/extension joint is implemented for each of hips. Height of the joint with respect to the torso brace is adaptable to allow manual alignment of rotation axes.

### 4.3. Motion Capturing

**Figure 7:**
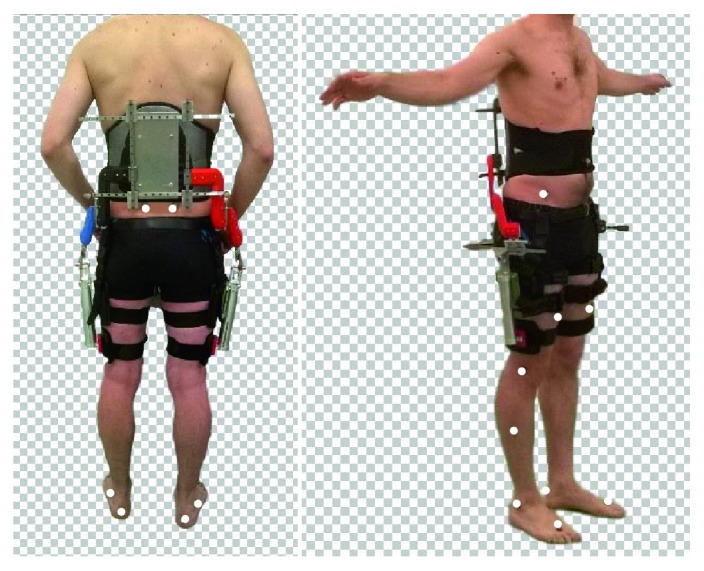
Placement of the markers according to the lower body plug-in-gait marker-set. Markers are placed on the pelvis (ASIS and PSIS), thigh, knee, shank, ankle, heel, and toe.

A sixteen-camera, infrared, optoelectronic, video-based motion analysis system at a sample rate of 100 Hz (Vicon Motion Systems, Oxford, UK) was used to record 3D movements of the lower limbs. Reflective markers were placed on specific anatomical landmarks, based on the marker protocol of the lower body Plug-in-gait marker-set, as shown in Figure 7. Five force plates (AMTI OR6 Series 1000 Hz) were used to capture ground interaction.

### 4.4. Experimental Protocol

**Figure 8:**
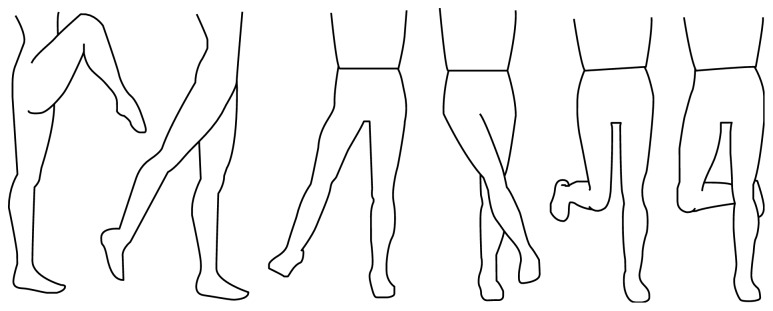
Poses to determine maximum movement amplitudes of the hip DOFs: maximum flexion, extension, abduction, adduction, and internal and external rotation.

The experimental protocol that is described in this section was executed three times by each subject: once for every equipment state. In order to be able to compare intrasubject data of the three executions, marker replacement was prevented. The subject was equipped with markers before test execution, and their placement was first checked for all three equipment states. The subject was then asked to adopt a pose of maximum movement amplitude for each of the hip DOFs, that is, maximum flexion, extension, abduction, adduction, internal rotation, and external rotation. Maximum movement amplitude poses for the right hip are shown in Figure 8. During the motion, the subject had to balance on one leg, without a stabilizing aid. Additionally, the subject was prompted to keep the pelvis and torso in its neutral position and only move the leg. The maximum poses were adopted five times each. First for flexion of the right hip, then following the order as given in Figure 8. Subsequently, this was repeated for the left hip. In between each movement, the subject was allowed to place both legs on the ground to regain balance if necessary. Lastly, the subject was asked to walk overground, in a straight line, at a self-selected walking speed, and this for a distance of approximately 10 m, delimited by a start and end marker. The subject was instructed to walk over the force places that were located halfway the trajectory.

### 4.5. Data Analysis

All positions were expressed in a coordinate system defined by the walking direction/face forward orientation (y), the vertical (z), and the axis perpendicular to this plane (x), according the right hand orientation. Marker labeling and trajectory reconstruction were performed using Nexus 2.5 (Oxford Metrics, UK) and filtered using Woltring filtering routine. Gait cycle events (i.e., initial contact and toe-off) were calculated from the force plate data.

To compare ROM for different equipment states per subject, maximum angles from the different poses were combined per plane of motion to get the full ROM per plane. Due to the absence of asymmetric behaviour in the unequipped state, data for left and right leg were combined, resulting in 10 data points per plane. It is expected that the data reveals that ROM in the PT state is the same as that in the UE state, but ROM in the 1D state differs from the latter. Because of the low number of samples (*N* = 10), *P* values were determined using the Wilcoxon signed rank test, where the null hypothesis stated that there was no difference visible in the ROM between the PT and UE state on one hand and between the 1D and UE state on the other.

The effect of the novel prototype and the 1-DOF module on the gait cycle should be directly reflected in the kinematics of the pelvis during walking. It is hypothesized that pelvic tilt, obliquity, and rotation amplitudes are unchanged while wearing the new prototype (with respect to the UE state), while they do change under the 1D condition. Indirectly, this should also be reflected in the step length and step width of the subject since pelvic rotation allows for a larger step length and smaller step width for the same flexion angle configuration [60]. Additionally, the effect of the equipment state is also expected to be visible in the fraction of double support during gait, as the double support phase of gait tends to lengthen when pelvic DOFs are blocked/hindered [61]. Double support fraction values were calculated by dividing the time in double support by the total stride time. In short, pelvic tilt, pelvic obliquity, pelvic rotation, step length, step width, and double support fraction are expected to change in the 1D condition, with respect to the UE state, while those in the PT state are expected to be equal to UE values. Because of the low number of samples, *P* values were determined using the Wilcoxon signed rank test, where the null hypothesis stated that, for each of the parameters, there was no difference between PT-UE and 1D-UE, respectively.

## 5. Results

### 5.1. Maximum ROM

**Figure 9:**
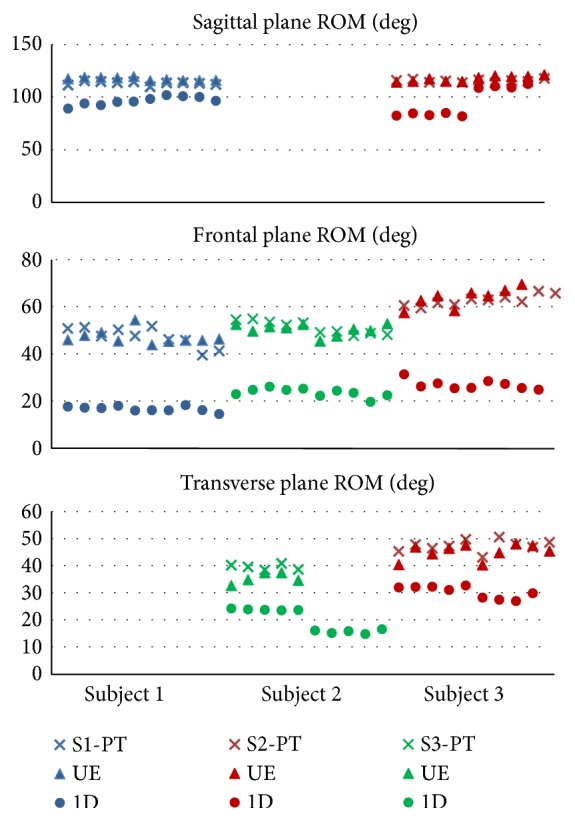
Maximum ROM measurements for all subjects in all equipment states. Subjects are denoted by color: subject 1 (S1) in blue, S2 in green, and S3 in red. The different equipment states are distinguished by marker symbol: crosses for the PT state, triangles for the UE state, and circles for the 1D state. The gaps in the graph are the result of data rejection due to marker disappearance and unauthorized movements of the torso during the testing procedure.

The results of the maximum hip ROM test are shown in Figure 9. Data is shown for all 3 equipment conditions. Maximum sagittal plane ROM for subject 2 is not available due to marker disappearance during the trial. For subject 1, data points in the transverse plane were rejected due to unauthorized movement of the torso. Similarly, for subject 2, five data points in the transverse plane were rejected for the UE and PT equipment state and two data points were rejected for subject 3 during measurement in the frontal plane while being unequipped.

Data rejection/unavailability, as described here, is responsible for the gaps in Figure 9.

The mean and median values of each data set are displayed in Table 2, together with the minima and maxima to give a measure of data spread. *P* values represent the probability that the median values of the PT and 1D states are equal to those in the UE state. The gaps in the table, for subjects 1 and 2, are the result of data rejection/unavailability as explained earlier. Differences in mean values for maximum ROM between the PT and UE states are situated between −4% and +2%. A negative value indicates a decrease with respect of the UE state, and a positive value indicates an increase. Only in the transverse trial of subject 2 a larger difference is seen, that is, +11.8%. Differences between the 1D and UE states are significantly larger, with values around −18% for the sagittal plane and between −32% and −65% for the other planes of motion.

### 5.2. Walking

The results of the recorded pelvis kinematics during gait are shown in Table 3. *P* values were calculated to test the hypothesis that median values of the PT and 1D trial do not differ from those in the UE trial. Pelvic tilt is not reported as it did not show any significant difference between states. The change of step length and step width between states was investigated, yet it is not reported here since no coherent trend was found between subjects. The fraction of double support time on the total stride time is displayed in Table 4, for each of the equipment states. *P* values were calculated, testing the equality of PT and 1D double support percentages with the UE values.

## 6. Discussion

In order to allow a subject to move completely unhindered, it is imperative that all human DOFs are allowed in an exoskeleton design. For the hip joint, those are flexion/extension, abduction/adduction, and internal/external rotation. Additionally, it is also required to ensure that corresponding human and exoskeleton joints are either aligned or that misalignment is compensated for. In order to verify that all human motions are allowed, a comparison of maximum allowed ROM for the different equipment states is considered to be a good start. Based on the allowed ROM and the required ROM for a certain activity of daily life, a conclusion could be made on the executability of that activity in each of the equipment states.

As shown in Table 2, allowed ROM of the 1-DOF prototype never equals the biological ROM in the unequipped state (*P* < 0.05), for any of the subjects. Mean values are consistently lower than the biological means, indicating that the 1-DOF module restricts the natural ROM of the hip joint. This result is to be expected in the frontal and transverse plane due to the absence of an abduction joint and a rotation joint in the module; however, the same is observed in the sagittal plane despite the presence of a flexion hinge. This observation contributes to the notion that misalignment of rotational axes influences the motion of the user. An extra observation strengthening this theory is the significant difference (*P* < 0.05, visible in Figure 9) of the 1D ROM between the left and right legs in the sagittal plane of both subjects. Since there is no significant difference in biological ROM between left and right legs for either subjects, one can only assume that a difference in alignment between both sides in the 1D case is the cause of this discrepancy. The same phenomenon is observed in the transverse plane for all subjects. As there is no rotation joint in the 1D module, misalignment is likely not the cause of the difference in this plane. Another valid explanation is found in the nature of the movement and the connection to the thigh. Due to the absence of a rotation joint, any motion in this plane is due to elastic deformation of the module and/or the soft tissue of the thigh. A difference in tightness of the straps around the thigh could possibly result in a different ROM, dependent on the comfort of the user. Reviewing the results, we reject the null hypothesis and thus accept the research hypothesis that ROM in the 1D and UE states is not equal to each other. Analysis of the performance of the prototype is more complex as it only exhibits statistical equality in the frontal plane for all subjects (*P* > 0.1) and the sagittal plane for subject 3 (*P* > 0.05). However, despite the low probability of equality in the transverse plane, it is interesting to note that the mean ROM in the PT state is actually higher than in the UE state for all data sets. Given that the prototype is purely passive, consisting only of hinges and linear sliders, it cannot be responsible for an increase in ROM. The difference in ROM in this situation is thus not clinically relevant. Only ROM in the sagittal plane for subject 1 shows a significant difference between PT and UE states (*P* < 0.05), despite the low percentual difference between both (3.5%). Although this is not the result that was expected, there is still a significant difference between ROM in the PT and 1D states (*P* < 0.05). The difference between means is 14.8%, indicating that the prototype still performs significantly better than the 1-DOF module. Additionally, it is important to note that there is no significant difference visible between right and left legs in the PT state, for any of the data sets. This is a clear indicator that misalignment is not influencing movement of the user while wearing the prototype. Given that only 1 data set acknowledges a difference between the UE and PT states, the research hypothesis that both states lead to equal ROM is accepted.

To investigate if maximum ROM results can be used to form conclusions on the executability of activities of daily life, overground walking was also examined. This is because of the high prevalence of walking over other tasks such as stair climbing and bending over. According to literature [62, 63], required hip ROM for level walking is 31°, 18°, and 16° for the sagittal, frontal, and transverse plane, respectively. Reviewing the maximum ROM results described above, ROM of both exoskeletons is sufficient to walk naturally. Other activities that require higher ROM, such as climbing stairs, are likely impossible while wearing the 1-DOF module. The influence of both exoskeletons on the gait cycle was researched by comparing pelvic motion, step length, step width, and double support fractions. Data concerning pelvic amplitudes are given in Table 3. Pelvic tilt is not shown as no differences were found between states. This is not unexpected as it relies on the presence of a flexion/extension joint, which is included in both exoskeletons, and ROM is small, reducing misalignment effects. Pelvic obliquity for the PT state is equal to that in the UE state for all subjects, but a significant change is seen between the UE and 1D states (*P* < 0.05). For pelvic rotation, a similar observation is made: motion values for the PT and UE states are equal and, apart from subject 2, motion values for the 1D state differ from the latter. Mean values for pelvic rotation in the 1D state are actually higher than those in the UE state, which is surprising. One would expect that the absence of a rotation joint in the design of an exoskeleton restricts motion in that direction, thus leading to a lower mean value. Although unexpected, such an increase is most likely to happen in the transverse plane, as the circular build and “meaty” structure of the upper leg allows for a significant amount of passive motion due to rotation of the thigh in the braces and/or deformation of the soft tissues. No coherent trend was found in step length and step width changes between equipment states for either of the subjects. The high dependence of both on other factors such as knee flexion angle and gait velocity is considered to be the cause of this observation. As mentioned in Section 4, the absence of an A axis and an R axis in the 1-DOF module is expected to have an influence of the amount of double support in the gait cycle. An increase is expected for the double support fraction in the 1D state, and no change is expected for the PT state. As seen in Table 4, double support fractions for the 1D state do show a significant difference with those of the UE state (*P* < 0.05), while those of the PT state are equal to UE values (*P* > 0.1). Only double support for subject 3 displays a deviation from UE values (*P* < 0.05). Despite the initial assumption that ROM of both exoskeletons was sufficient to allow natural gait, pelvic motions and double support times displayed significant differences in the 1D condition. Clearly, an evaluation of the allowed ROM, while wearing a device, alone is not sufficient to draw conclusions on its influence on natural motion patterns. A possible explanation for this observation during gait lies in the manner in which the ROM is achieved. Due to the absence of an A axis and an R axis in the 1-DOF module, rotation around the corresponding biological axes is the result of passive deformations of the device and/or soft tissue of the user. Deformation of these structures requires force input that needs to be provided by the user's muscles, thus changing joint torques. Earlier research indicates that when wearing assistive devices, humans adapt their gait strategy in order to maintain global joint torque trajectories rather than joint kinematics [64, 65]. This could explain the adapted kinematics witnessed while analysing 1-DOF exoskeleton gait. The lack of difference in PT kinematics, compared to the UE state, could be an indication that natural muscle activity patterns are maintained. This is a valid assumption because the presence of all 3 rotation axes in the prototype eliminates the need for elastic deformation to maintain natural ROM. Further research, including measurement of muscle activity through EMG tracking, is required to confirm this statement. These measurements can also serve as a tool to explain why pelvic rotation, wearing the 1-DOF module, did not decrease as expected but rather increased and as such provides an insight into the actual mechanisms of exoskeleton gait adaptation.

## 7. Conclusion

The presented work introduced a novel full-DOF hip exoskeleton, with misalignment compensation for all DOFs. Extensive testing of the prototype has shown that maximum ROM of the user is maintained while wearing the device.

No difference in left or right leg kinematics was displayed in either of the tests, indicating that effects of misalignment were successfully compensated. Although the results shown for the 1-DOF module call for caution when maximum ROM measurements are used to draw conclusions for other motion patterns, no difference in the investigated gait parameters was visible after donning the prototype. This is probably due to the fact that full ROM was maintained in the PT state, whereas only partial ROM was maintained in the 1D state, which was achieved by elastic deformation. This leads us to believe that other activities in the PT state would remain uninfluenced as well, although this needs to be verified in an additional study. The exoskeleton is designed to limit the mass and number of components. The misalignment compensation mechanism ensures an equal change in angles of the human and the exoskeleton, facilitating easy actuation of the prototype in a further stage and limiting the amount of required sensors. The components that are used are low cost and do not require regular maintenance. Donning of the device is easy, starting with attachment of the belt around the torso and followed by tightening of the straps around the thighs. The position of the straps on the thigh can be altered for maximum comfort. This adaptation only needs to be performed once. Adaptation to anatomy of the user is done automatically, as this only results in a change of the rest position of the sliders while donning. This mechanism also copes with slight differences in brace position on the body between trials, eliminating the need for recurring adaptations and significantly reducing donning times. Slipping of the braces on the thigh is prevented by an attachment to the user's belt. Donning can be performed autonomously. This is further ameliorated by the addition of shoulder straps in the following version of the prototype, allowing the user to put it on like a backpack.

## 8. Future Work

Work in the current paper has shown that the prototype is not hindering its wearer during walking. The maximum ROM study hints that this is likely also the case for other activities of daily life, although this needs to be verified in another kinematic study. The focus of future work, however, will be the reduction of metabolic cost of the user during walking and sit to stand activities. The kinematic prototype, presented in this work, is designed in such a way that it is able to transmit a torque of 20 Nm from an actuator to the user. Thus, for future work, it only needs to be fitted with an actuation system that can assist the user during the tasks mentioned earlier. The actuation system should be lightweight as it will be located at the flexion joint on the thigh of the subject. Due to the intended use of the device, that is, assistance during daily life, considerable importance will be attached to untethered operation capabilities. Use of inherently compliant actuators appears to be a promising strategy, due to the advantages in safety and energy consumption [66]. Past experience with exoskeleton actuation and control points towards the implementation of a MACCEPA-based compliant actuator with adaptable compliance as a suitable means for actuating the novel prototype. More info on the MACCEPA and instrumentation for efficient compliant actuator control can be found in [2] and [67], respectively.

## Figures and Tables

**Table 1:** Subject data.

Subject	S1	S2	S3
Sex (M/F)	M	M	M
Age	30	29	25
Height (m)	1.85	1.90	1.79
Weight (kg)	77.9	91.3	69.9

**Table 2:** Mean, median, minimum, and maximum values of hip ROM for each of the 3 equipment states. Sagittal plane data is not available for subject 2 due to marker disappearance. Transverse plane data for subject 1 was rejected due to unauthorised movement of the torso. ^∗^*P* values are unitless and reflect the probability that the median value of the prototype (PT) and 1-DOF (1D) trial does not differ from that in the unequipped (UE) trial. As *P* values reflect the result of a comparison with the UE state data, and they are not relevant (N.R.) for the UE state itself.

		Sagittal ROM (°)				Frontal ROM (°)				Transverse ROM (°)			
		Mean	Median	*P* value^∗^	Min/max	Mean	Median	*P* value^∗^	Min/max	Mean	Median	*P* value^∗^	Min/max
S1	PT	113.3	113.6	<0.05	109.8/115.6	47.2	47.6	>0.1	39.5/51.7	—	—	—	—
	UE	117.4	117.1	N.R.	115.7/119.6	47.0	45.9	N.R.	43.8/54.4	—	—	—	—
	1D	96.5	96.2	<0.05	89.2/102.0	16.7	16.6	<0.05	14.5/18.3	—	—	—	
S2	PT	—	—	—	—	50.8	50.9	>0.1	47.7/54.9	39.5	39.6	<0.05	38.4/40.8
	UE	—	—	—	—	50.1	50.8	N.R.	45.4/52.8	35.3	34.8	N.R.	32.6/37.4
	1D	—	—	—	—	23.7	23.9	<0.05	19.7/26.1	19.7	20.0	<0.05	14.7/24.2
S3	PT	115.5	115.7	0.05 < *P* < 0.1	113.5/117.8	72.7	61.9	>0.1	59.5/64.0	47.4	47.5	<0.05	43.1/50.6
	UE	117.6	118	N.R.	114.2/121.2	73.7	64.7	N.R.	57.5/69.5	45.1	45.9	N.R.	40.3/48.0
	1D	95.3	85.0	<0.05	81.8/112.5	26.9	26.7	<0.05	25.4/31.3	30.4	30.4	<0.05	26.0/32.6

**Table 3:** Mean, median, minimum, and maximum values of pelvic obliquity and pelvic rotation amplitudes during walking for each of the subjects in each of the 3 equipment states. ^∗^*P* values are unitless and reflect the probability that the median value of the prototype (PT) and 1-DOF (1D) trial does not differ from that in the unequipped (UE) trial. As *P* values reflect the result of a comparison with the UE state data, they are not relevant (N.R.) for the UE state itself.

	Pelvic obliquity (°)				Pelvic rotation (°)			
	Mean	Median	*P* value^∗^	Min/max	Mean	Median	*P* value^∗^	Min/max
PT	2.4	2.8	>0.1	1.5/3.3	5.0	4.4	>0.1	1.5/9.4
S1 UE	2.5	2.6	N.R.	1.6/3.6	5.5	5.8	N.R.	0.1/8.9
1D	2.0	2.0	<0.05	0.8/2.6	7.5	8.0	*P* = 0.1	2.4/9.9
PT	4.9	5.9	>0.1	1.8/7.4	6.7	6.6	>0.1	4.2/10.7
S2 UE	5.8	6.2	N.R.	4.1/8.0	6.3	6.1	N.R.	3.0/10.9
1D	2.3	2.4	<0.05	1.8/2.9	7.3	7.1	>0.1	4.3/10.0
PT	5.3	4.9	>0.1	4.0/5.8	4.5	5.4	>0.1	1.5/6.9
S3 UE	5.7	4.2	N.R.	3.3/8.7	4.2	4.2	N.R.	3.1/6.2
1D	3.5	2.9	<0.05	2.4/4.9	7.1	6.4	<0.05	5.1/9.8

**Table 4:** Mean, median, minimum, and maximum values of the fraction of double support in the gait cycle. ^∗^*P* values are unitless and reflect the probability that the median value of the prototype (PT) and 1-DOF (1D) trial does not differ from that in the unequipped (UE) trial. As *P* values reflect the result of a comparison with the UE state data, they are not relevant (N.R.) for the UE state itself.

		Double support (% of ST)			
		Mean	Median	*P* value^∗^	Min/max
	PT	10.9	10.6	>0.1	10.0/12.8
S1	UE	10.1	10.1	N.R.	8.3/12.9
	1D	12.4	12.4	<0.05	11.3/13.1
	PT	10.1	10.5	>0.1	7.8/11.8
S2	UE	9.9	10.0	N.R.	8.6/10.7
	1D	10.9	10.8	<0.05	10.2/12.5
	PT	9.8	9.5	<0.05	8.2/13.8
S3	UE	7.3	6.8	N.R.	5.9/10.1
	1D	9.4	9.2	<0.05	8.1/11.1
